# Angiopoietin-Like 4 Mediates PPAR Delta Effect on Lipoprotein Lipase-Dependent Fatty Acid Uptake but Not on Beta-Oxidation in Myotubes

**DOI:** 10.1371/journal.pone.0046212

**Published:** 2012-10-04

**Authors:** Marius R. Robciuc, Paulina Skrobuk, Andrey Anisimov, Vesa M. Olkkonen, Kari Alitalo, Robert H. Eckel, Heikki A. Koistinen, Matti Jauhiainen, Christian Ehnholm

**Affiliations:** 1 Department of Chronic Disease Prevention, National Institute for Health and Welfare, Helsinki, Finland; 2 Unit of Metabolism, Minerva Foundation Institute for Medical Research, Helsinki, Finland; 3 Department of Medicine, Division of Cardiology, Helsinki University Central Hospital, Helsinki, Finland; 4 Institute of Clinical Medicine, Faculty of Medicine, University of Helsinki, Helsinki, Finland; 5 Molecular/Cancer Biology Laboratory, Institute for Molecular Medicine Finland, Research Programs Unit and Department of Pathology, University of Helsinki, Helsinki, Finland; 6 Institute of Biomedicine, Anatomy, University of Helsinki, Helsinki, Finland; 7 Division of Endocrinology, Metabolism, and Diabetes, Department of Medicine, University of Colorado, Anschutz Medical Campus, Aurora, Colorado, United States of America; Wageningen University, Netherlands

## Abstract

Peroxisome proliferator-activated receptor (PPAR) delta is an important regulator of fatty acid (FA) metabolism. Angiopoietin-like 4 (Angptl4), a multifunctional protein, is one of the major targets of PPAR delta in skeletal muscle cells. Here we investigated the regulation of Angptl4 and its role in mediating PPAR delta functions using human, rat and mouse myotubes. Expression of Angptl4 was upregulated during myotubes differentiation and by oleic acid, insulin and PPAR delta agonist GW501516. Treatment with GW501516 or Angptl4 overexpression inhibited both lipoprotein lipase (LPL) activity and LPL-dependent uptake of FAs whereas uptake of BSA-bound FAs was not affected by either treatment. Activation of retinoic X receptor (RXR), PPAR delta functional partner, using bexarotene upregulated Angptl4 expression and inhibited LPL activity in a PPAR delta dependent fashion. Silencing of Angptl4 blocked the effect of GW501516 and bexarotene on LPL activity. Treatment with GW501516 but not Angptl4 overexpression significantly increased palmitate oxidation. Furthermore, Angptl4 overexpression did not affect the capacity of GW501516 to increase palmitate oxidation. Basal and insulin stimulated glucose uptake, glycogen synthesis and glucose oxidation were not significantly modulated by Angptl4 overexpression. Our findings suggest that FAs-PPARdelta/RXR-Angptl4 axis controls the LPL-dependent uptake of FAs in myotubes, whereas the effect of PPAR delta activation on beta-oxidation is independent of Angptl4.

## Introduction

Skeletal muscle is the main site for burning excess calories when exercised hence, it plays a key role in the prevention and management of metabolic diseases such as type 2 diabetes. In our modern society there is a chronic positive energy balance due to overnutrition and inactivity. An oversupply of skeletal muscle with fatty acids (FAs), exceeding its oxidative capacity, results in accumulation of intramyocellular lipids. Increased ectopic lipid deposition in liver and skeletal muscle is associated with increased concentrations of active lipid metabolites such as diacylglycerols and ceramides, impaired insulin signaling, and thus plays a major role in lipid-induced insulin resistance of obesity and type 2 diabetes [1]. A deeper understanding of the mechanisms regulating FA uptake in skeletal muscle can potentially be used to ameliorate perturbations in systemic lipid balance and insulin sensitivity.

Skeletal muscle, as well as other tissues, obtain FAs either via lipoprotein lipase (LPL, EC 3.1.1.34)-mediated hydrolysis of lipoprotein triglycerides or from unesterified FAs bound to albumin [2]. Although it is difficult to discern which is the major pathway for FA uptake, the LPL-mediated uptake has the advantage of being tightly regulated in a tissue specific manner independent of albumin-bound FAs. Stored FAs are released from adipose tissue by intracellular lipolysis and are rapidly taken up by the liver, esterified to triglycerides, packed in lipoproteins and secreted as VLDL. Dietary FAs are absorbed by enterocytes, packed and secreted into the circulation as chylomicrons. Thus, >90% of circulating FAs are contained within VLDL and chylomicron particles and delivered to skeletal muscle and heart (oxidation) or adipose tissue (storage) by LPL-catalyzed lipolysis [2]. LPL is produced in parenchymal cells of these tissues, and it is transported and bound by glycosylphosphatidylinositol-anchored high density lipoprotein-binding protein 1 (GPIHBP1) to the luminal side of the capillary endothelium where it exerts its main function [3], [4].

Peroxisome proliferator-activated receptor (PPAR) δ is the main PPAR isoform in skeletal muscle and an important regulator of FA metabolism [5]–[10]. Due to their capacity to enhance FA catabolism specific PPARδ agonists, such as GW501516, are promising drugs to treat metabolic diseases such as type 2 diabetes. Interestingly, one major target of PPARδ in skeletal muscle is angiopoietin-like 4 (Angptl4) [11], a potent inhibitor of LPL [12], [13]. In addition, Angptl4 is a modulator of adiposity by enhancing intracellular lipolysis in adipocytes [14]–[16].

Here we describe the mechanism by which PPARδ activation by FAs upregulates Angptl4 and inhibits LPL activity and LPL-mediated FA uptake in myotubes. Since Angptl4 is a multifunctional protein we also tested its effect on FA oxidation and glucose metabolism.

## Materials and Methods

### Cell Culture

Muscle samples for primary human myoblast cultures were obtained from surgical open muscle biopsies. Biopsies were taken from 6 nondiabetic men (BMI 25.1±4.2 kg/m^2^ (mean ± SD), age 54±7 yrs). Satellite cells were isolated, myoblasts proliferated and differentiated into myotubes as previously described [17]. Written informed consent was obtained from the subjects and the study was approved by the Ethical Committee of Department of Medicine, Helsinki University Central Hospital. All experiments were performed in accordance with the principles of the Declaration of Helsinki, as revised in 2000. L6 (ATCC, CRL-1458) and C2C12 (ATCC, CRL-1772) myoblast cell lines were maintained in high glucose Dulbecco's modified Eagle's medium (DMEM) supplemented with 10% (v/v) fetal calf serum, 10 mM HEPES, pH 7.4, 100 U/mL penicillin, and 100 µg/mL streptomycin (full growth medium). Details about C2C12 myoblasts stably transfected with human LPL (C2/LPL) were described previously [18]. When cells reached 60–80% confluence full growth medium was replaced with DMEM containing 2% horse serum (differentiation medium) to induce the differentiation of proliferating myoblasts. Differentiation medium was replaced every day for 6–7 days until multinucleated myotubes were formed. All experiments have been performed in differentiation medium unless otherwise specified or required by previously described protocols. In experiments where BSA-bound oleic acid (OA-BSA) was used defatted BSA at the same concentration was added in control samples and combined with other treatments. Conventional light microscopy images were collected during the differentiation process. Human myotubes were fixed and stained with hematoxylin before image collection. The viability and attachment of the cells were carefully evaluated by light microscopy and protein measurements and no cytotoxic effects could be observed throughout the study.

### Preparation and in vitro delivery of recombinant adeno-associated viruses (AAV)

Human Angptl4 (hAngptl4) and serum albumin (HSA) cDNAs were cloned into blunted MluI and NheI restriction sites of the psubCMV-WPRE recombinant AAV expression vector [19]. The AAVs serotype 2 and 9 (AAV2 and AAV9) were produced as described previously [20]. AAV2 was more efficient in transducing cells in vitro compared to AAV9 whereas the opposite was true in vivo. Virus transduction was performed on day four of differentiation using Angptl4-AAV2 or HSA-AAV2 at a concentration of 16×10^9^ virus genomes per ml. Experiments were performed 48 or 72 hours post-infection.

### Quantitation of Angptl4 by ELISA

The development and validation of hAngptl4 ELISA was published previously [21]. Quantification of secreted hAngptl4 was performed in medium (with or without serum) after a twofold dilution with the ELISA dilution buffer [21]. To measure cell associated hAngptl4 myotubes were lysed in RIPA buffer [50 mM Tris, pH 8.0, 150 mM NaCl, 1% (v/v) NP-40, 0.5% (v/v) sodium deoxycholate, 0.1% SDS] supplemented with complete mini EDTA-free protease inhibitor cocktail (Roche). Cell lysate was diluted to a minimum of four fold without significant effect on the ELISA measurements.

### Gene expression analyses

Angptl4 mRNA levels were measured by real time PCR. Cultured cells were homogenized in RLT buffer (Qiagen) and total RNA was isolated with RNeasy Mini kit (Qiagen) according to the manufacturer's instructions. The total RNA (2 µg) was reverse-transcribed using Maxima First Strand cDNA Synthesis Kit (Fermentas). Samples were amplified on a 7000 Sequence Detection System (Applied Biosystems), using FastStart Universal SYBR Green Mastermix with ROX (Roche). Housekeeping genes used were βActin and PBGD (human) or 36B4 and HPRT (mouse). Primer sequences are provided in the Table S1.

### RNA interference

C2/LPL myoblasts were seeded in 24-well plates and transfected with siGENOME siRNA set (Dharmacon, Thermo Scientific) at 25 pmols/well as previously described [11] with minor modifications. Transfection was achieved using DharmaFECT1 (Dharmacon, Thermo Scientific) and cells were allowed to differentiate only for 3 days before LPL activity was assayed due to the transient nature of siRNA silencing.

### SDS-PAGE and Immunoblot Analysis

C2/LPL myotubes were differentiated in 12-well plates and incubated in 0.5 ml serum free medium per well. Conditioned media were concentrated approximately 100-fold using Nanosep 10k Omega columns (Pall) and equal volumes, corresponding to 200 µl of non-diluted medium, were separated on a NuPAGE 4–12% Novex Bis-Tris gels (Invitrogen). Proteins were electro-transferred to nitrocellulose membranes and the transfer quality was evaluated by Ponceau red staining. Endogenous mouse Angptl4 (mAngptl4) secreted from C2/LPL cells was detected using a purified polyclonal rabbit IgG raised against recombinant mAngptl4 (AG-25A-0071-C100, Adipogen). As a positive control we used medium from HEK293 cells transfected with recombinant mAngptl4, using a plasmid kindly provided by Professor Aimin Xu (University of Hong Kong, China).

### LPL Activity

L6 and C2C12 myotubes were differentiated in 10-cm plates whereas C2/LPL myotubes were differentiated in 6-well, 12-well or 24-well plates. Plates were transferred on ice, medium was aspirated and cells were washed three times with cold PBS. LPL was released from myotubes by incubating the cells with Krebs Ringer Hepes buffer (130 mM NaCl, 10 mM HEPES, 10 mM MgSO_4_×7H_2_O, 2.5 mM NaH_2_PO_4_×H_2_O, 4.6 mM KCl, 2.5 mM CaCl_2_×2H_2_O, 2.5 mM Na-pyruvate, pH 7.4) containing 100 µg/ml Heparin (KRH-Heparin) for 5 minutes on ice (heparin releasable LPL). To measure intracellular LPL activity cells were further washed with PBS and incubated with Trypsin-EDTA [0.1% (w/v) Trypsin, 50 mM EDTA in PBS, pH 7.4] at room temperature (RT) for approximately 5 minutes, until cells detached. Further, cells were transferred back on ice and Trypsin-EDTA removed by washing twice with cold PBS. The pellet was centrifuged at 300 g for 3 minutes at +4°C and sonicated for 10 seconds in KRH-Heparin buffer (intracellular LPL). Heparin releasable and intracellular LPL activity was measured as previously described [22] with some modifications. Briefly, [Carboxyl-^14^C]-Triolein (S.A. 2.2 GBq/mmol, PerkinElmer) and glyceryl trioleate (Sigma) emulsified in the presence of gum arabic was used as substrate. KRH-Heparin buffer derived from myotubes (heparin releasable or intracellular LPL) was incubated with the substrate and human serum (as a source for apoC-II, LPL cofactor) for 1 hour at 37°C. Reaction was stopped by addition of 3.25 ml of methanol-chloroform-heptane (1.41∶1.25∶1.00, vol/vol/vol) and 0.75 ml of potassium carbonate/borate buffer (pH 10.5). Hydrophilic phase (BSA-FAs) and hydrophobic phase (triolein) were separated by centrifugation and radioactivity was measured from both fractions by liquid scintillation counting (Wallac LS Counter, Turku, Finland). LPL activity was expressed as mol of FAs released per hour per mg of cell protein. To evaluate the effect of purified Angptl4 on LPL activity we used recombinant hAngptl4 (4487AN, R&D Systems).

### Oil Red O staining

To quantify and visualize the intracellular neutral lipids we used Oil Red O (ORO) staining. Myotubes were differentiated in 6-well, 12-well or 24-well plates with or without coverslips and incubated with lipids for 5 (C2/LPL) or 16 (L6) hours. OA-BSA or Intralipid (Sigma) were added 48 hours after infection or 2 hours after treatment with the LPL inhibitor Orlistat (Sigma). After fixation with 10% formalin in PBS myotubes were stained with 0.12% (w/v) ORO in 60% isopropanol. Myotubes were thoroughly washed, ORO was extracted with 100% isopropanol and absorbance was measured at 490 nm. Empty wells were used to measure the blank. For cells grown on coverslips nuclei were stained with DAPI, mounted and visualized in fluorescence microscopy using Axioplan 2 Imaging E (Zeiss) microscope.

### Triglyceride quantification

To quantify the intracellular triglycerides in C2/LPL myotubes cells were lysed in RIPA buffer, sonicated and then heated at 95°C for 10 minutes until samples became cloudy. After the samples reached room temperature insoluble cell debris was removed by centrifugation and triglycerides were measured using an enzymatic colorimetric assay (Cobas, Roche/Hitachi).

### Fluorescence microscopy

C2/LPL myoblasts seeded on coverslips were fixed with 4% paraformaldehyde, PBS, pH 7.4 for 20 min at RT. Permeabilisation and blocking was performed with 10% heat inactivated FBS in PBS with 0.1% Triton X-100 for 1 hour at RT. The coverslips were incubated with primary antibodies in blocking buffer at 4°C, overnight. Coverslips were washed 3×5 min with PBS, 0.1% Tx-100 to remove unbound antibodies and incubated with the secondary antibodies (Alexa 549- or Alexa 633-labelled goat anti chicken) diluted 1∶200 in blocking buffer for 1 hour at RT. After washing 3×5 min with PBS, 0.1% Tx-100, the specimens were mounted in ProLong Gold (Invitrogen) and analyzed using a Leica TCS SP2 laser scanning confocal microscope system. LPL was detected using an immunopurified chicken antibody against LPL (a gift from Professor Gunilla Olivecrona, Umeä, Sweden) diluted at 7 µg/ml with blocking buffer. For co-localization studies C2/LPL myoblasts were transfected with human Angptl4 using a plasmid kindly provided by Professor Sander Kersten (Wageningen University, The Netherlands) and Lipofectamine 2000 (Invitrogen). After immunostaining for LPL, Angptl4 was detected with an anti-V5 Mouse mAb, FITC Conjugate (Invitrogen) diluted 1∶500 in blocking buffer for 1 hour at RT. The specificity of the antibodies was tested using mock transfected cells (for Angptl4) or by pre-incubating the antibodies with bovine LPL (for LPL).

### Fatty acid oxidation

L6 myotubes were differentiated in 6-well or 12-well plates and FA oxidation was measured using [1-^14^C]-Palmitic acid (S.A. 2 GBq/mmol, ARC 0172A, ARC) or [9,10-^3^H(N)]-Palmitic acid (S.A. 1.5 TBq/mmol, ART 0129, ARC) 72 hours post-infection. [1-^14^C]-Palmitic acid oxidation to ^14^C-CO_2_ and ^14^C-Acids soluble metabolites (ASM) was measured as previously described [23] with some modifications. Briefly, cells were incubated with 0.4 µCi per well (1 ml) of BSA-bound palmitic acid in serum free DMEM (5 mM glucose) for 90 minutes. Medium (750 µl) was transferred in 2 ml tubes containing 150 µl 70% percloric acid and having Whatman paper soaked in 30 µl 5N NaOH placed in the caps. Caps were closed quickly avoiding contamination of the Whatman paper with radioactive medium. The CO_2_ was released from the media and trapped on the Whatman paper by incubating the tubes for 90 minutes at 37°C. [9,10-^3^H(N)]-Palmitic acid oxidation to ^3^H-H_2_O was measured as previously described [24] after four hours of incubation in the presence or absence of GW501516. Labeled CO_2_, ASM and H_2_O were measured by liquid scintillation counting (Wallac LS Counter, Turku, Finland) and values expressed as µmol of FA oxidized per minute per mg of cell protein.

### Glucose uptake, glycogen synthesis and glucose oxidation

L6 myotubes were differentiated in 12-well plates and analyzed 72 hours post-infection. Glucose uptake was measured using 2-[1,2-^3^H(N)]-Deoxy-D-glucose (2DG, S.A. 250 GBq/mmol, NET328A001MC, Perkin Elmer) as described previously [25]. Glycogen synthesis and glucose oxidation were measured in the same experiments using D-[^14^C(U)]-Glucose (S.A. 10 GBq/mmol, ARC 0122H, ARC) in 5 mM glucose DMEM. Incorporation of glucose into glycogen was measured after isolation of glycogen as described previously [17]. Oxidation of glucose to CO_2_ was measure by adapting the protocol described for palmitate oxidation [23]. Briefly, cells were incubated cells were incubated with 0.5 µCi per well (0.5 ml) of D-[^14^C(U)]-Glucose in serum free DMEM (5 mM glucose) for 90 minutes. Medium (400 µl) was transferred in 2 ml tubes containing 75 µl 70% percloric acid and having Whatman paper soaked in 30 µl 5N NaOH placed in the caps. Caps were closed quickly and the CO_2_ was released from the media and trapped on the Whatman paper by incubating the tubes for 90 minutes at 37°C. Radioactivity was measured by liquid scintillation counting (Wallac LS Counter, Turku, Finland) and values were expressed as pmol of glucose per minute per mg of cell protein.

### Statistical analyses

Values from at least 3 experiments are reported as mean ± SEM and statistical significance was determined using paired *t* test or One- or Two-way ANOVA, as specified in each figure. Values of p<0.05 were considered as significant. Statistical analyses were performed using GraphPad Prism for Windows, GraphPad Software, San Diego California USA, www.graphpad.com.

## Results

### Angptl4 mRNA and protein levels are increased during differentiation of myotubes

**Figure 1:**
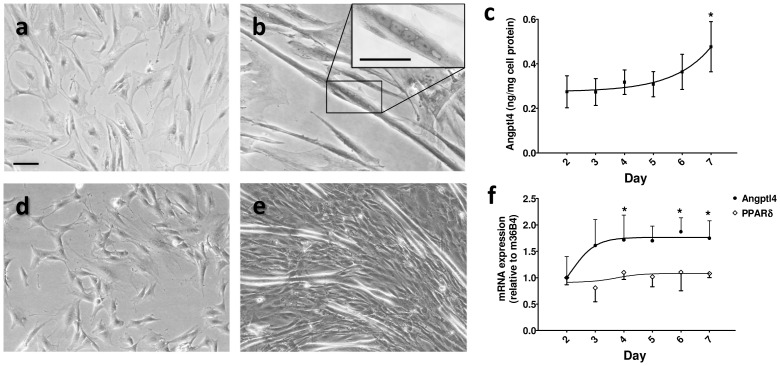
Angptl4 mRNA and protein levels are increased during differentiation of myotubes. Proliferative myoblasts from human (**a**) and mouse (**d**) C2/LPL were differentiated in multinucleated (**b**, inset) myotubes (**b**, human and **e**, mouse) and visualized using conventional light microscopy. (**c**) Secretion of Angptl4 was quantified by ELISA from day 2 to day 7 of differentiation and adjusted for protein content in cells derived from three men. (**f**) Angptl4 and PPARδ mRNA levels were measured by real time PCR during differentiation of C2/LPL myoblasts, n = 4. Values are expressed as fold increase relative to day two of differentiation and normalized to human β-actin or mouse 36B4 mRNA levels. Results are expressed as mean ± SEM, (**c**) n = 3 and (**f**) n = 4. Scale bar: 50 µm. *p<0.05 as compared with day 2 using Repeated Measures ANOVA with Dunnett's post test.

To study the Angptl4 expression during differentiation of myoblasts into myotubes we used human (Fig. 1a, b) and mouse C2/LPL (Fig. 1d, e) cells. Proliferative myoblasts (Fig. 1a, d) were differentiated for seven days to form multinucleated myotubes (Fig. 1b, e). A progressive increase of Angptl4 secretion during differentiation of human cells was observed (Fig. 1c). This is supported by a significant increase of Angptl4 mRNA levels on day 5 and 7 when compared with day 2 of differentiation, whereas PBGD mRNA levels remained unchanged (Fig. S1). In mouse C2/LPL cells we observed an increase of Angptl4 mRNA levels during the first three days of differentiation before reaching a plateau level on day four. In contrast, the mRNA levels of *Pparδ* (Fig. 1f) and the housekeeping gene, HPRT, (data not shown) were not changed during the differentiation process. Secretion of Angptl4 from mouse cells could not be measured by Western blot due to very low protein levels and presence of serum in the differentiation medium.

### Regulation of Angptl4 expression in myotubes

**Figure 2:**
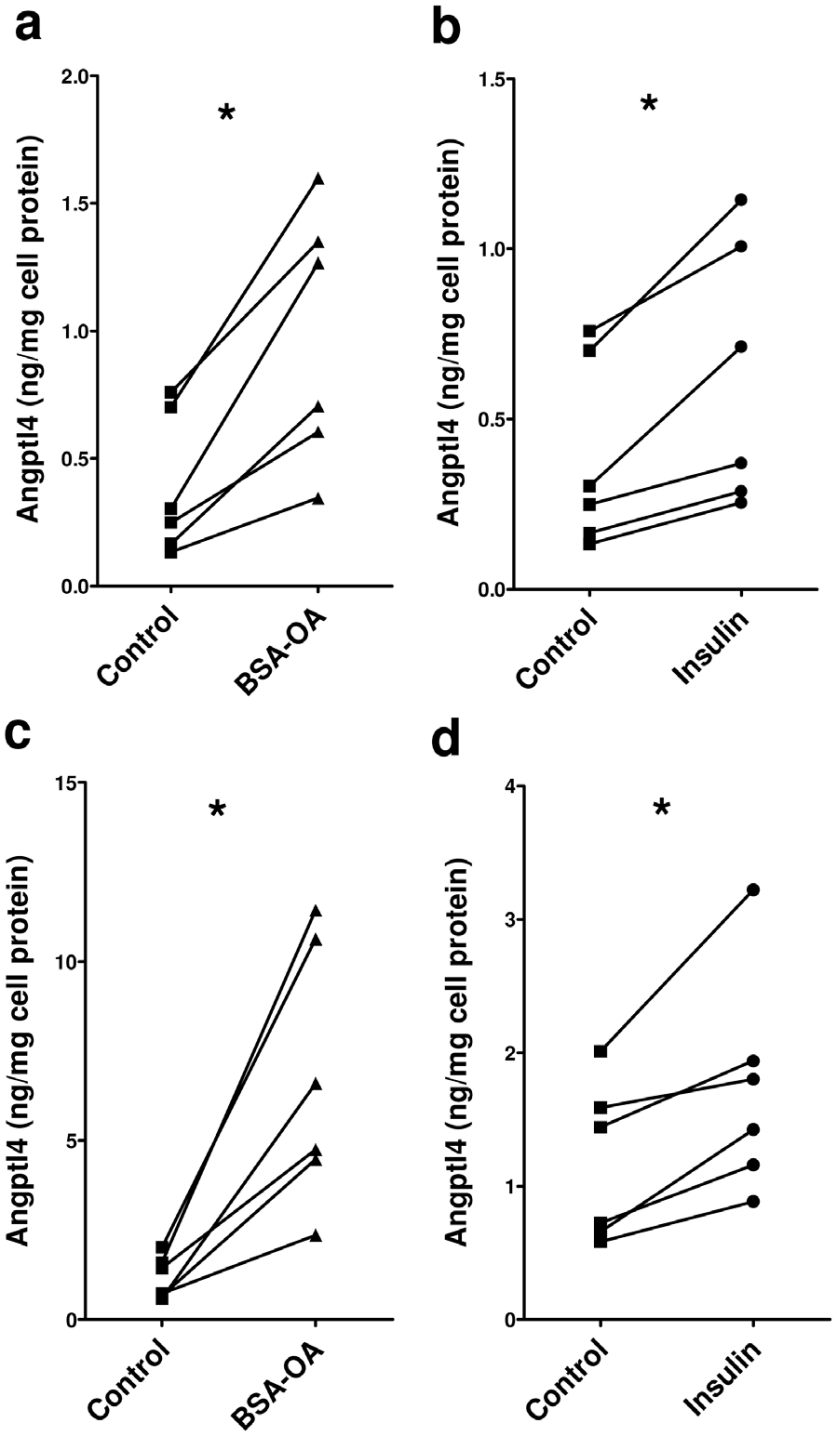
Angptl4 protein expression is increased by free fatty acids and insulin in human myotubes. Concentration of (**a, b**) cell associated and (**c, d**) secreted Angptl4 protein was quantified by ELISA in myotubes derived from six men and incubated in the presence of (**a, c**) BSA-bound oleic acid (OA-BSA, 0.5 mM) or (**b, d**) Insulin (100 nM) for 24 hours. *p<0.05, paired *t* test compared with Control.

In order to study the regulation of hAngptl4 by OA-BSA and insulin we used fully differentiated myotubes derived from six healthy men. An increase in cell associated (Fig. 2a, b) and secreted Angptl4 (Fig. 2c, d) was observed when myotubes were incubated in the presence of OA-BSA or insulin for 24 hours.

**Figure 3:**
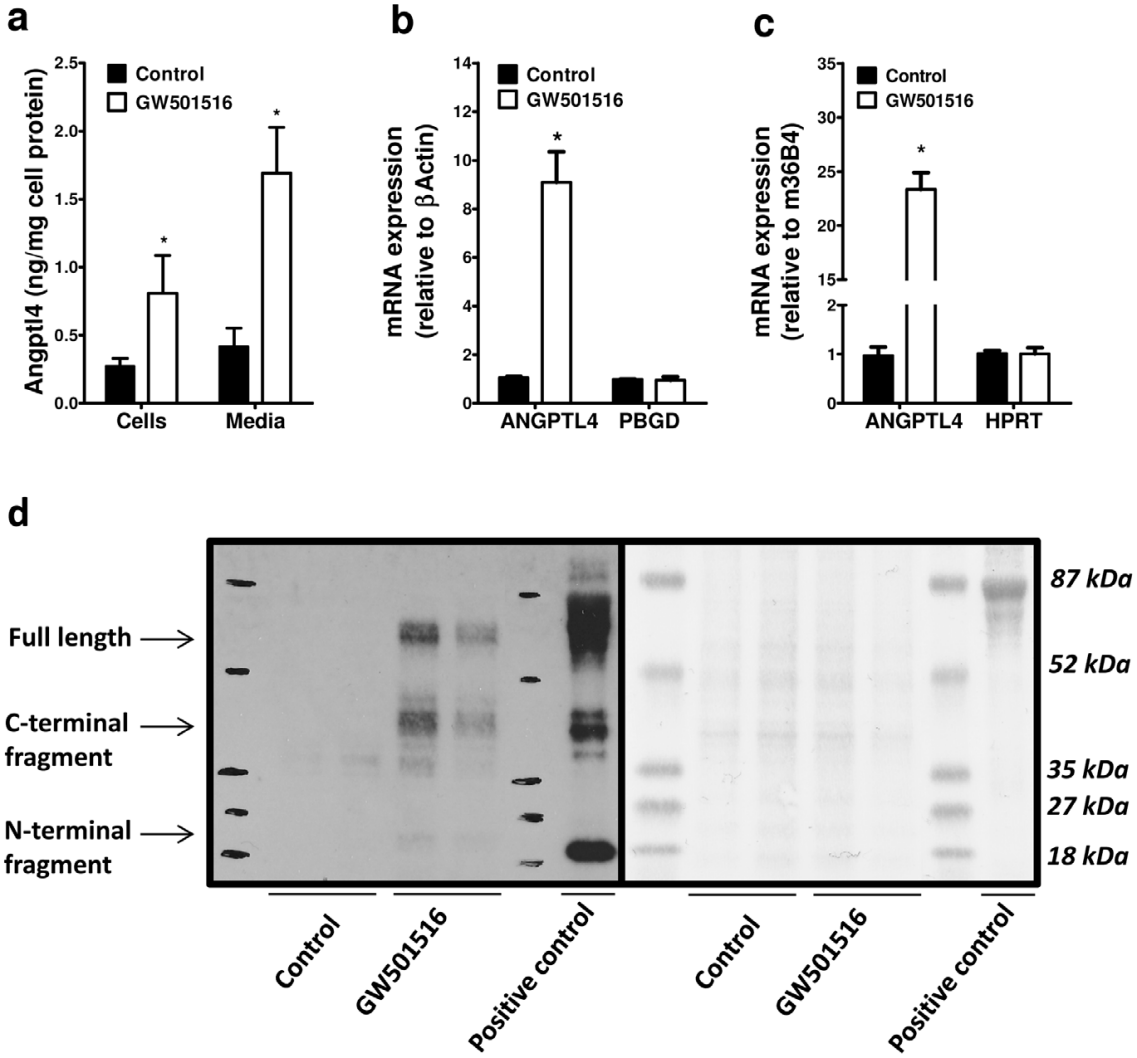
PPARδ activation increases Angptl4 mRNA and protein levels in human and mouse C2/LPL myotubes. (**a**) Human myotubes were incubated for 24 hours in the presence of DMSO (Control) or GW501516 (0.1 µM) and Angptl4 concentration in medium and cell lysate was quantified using ELISA, n = 4. Angptl4 mRNA levels were measured by real time PCR in (**b**) human and (**c**) mouse myotubes incubated with DMSO (Control) or GW501516 (0.1 µM) for 24 hours, n = 4. Angptl4 mRNA levels were normalized and analyzed in parallel with human PBGD and mouse HRPT mRNA levels. (**d**) Secretion of Angptl4 from C2/LPL myotubes was analyzed by Western blot 48 hours after incubation with DMSO (Control) or GW501516 (0.1 µM). Samples from two experiments were loaded on the gel. Right panel shows Ponceau staining of the blotting membrane as a control for protein loading and efficient transfer from the gradient gel to nitrocellulose membrane. *p<0.05, paired *t* test compared with Control.

Using GW501516, a synthetic PPARδ agonist, we obtained a similar induction of Angptl4 expression in human myotubes as observed with OA-BSA (Fig. 3a). Treatment of myotubes with GW501516 for 24 hours induced a robust increase in Angptl4 mRNA levels in both human and mouse C2/LPL cells (Fig. 3b and c respectively). Secretion of endogenous mAngptl4 was detected by Western blot only from C2/LPL cells stimulated with GW501516 (Fig. 3d). Using an antibody that recognizes all 3 forms of mAngptl4 we were able to detect the full-length, C-terminal, and N-terminal fragments of both endogenous and overexpressed protein (Fig. 3d).

### PPARδ activation inhibits LPL activity and LPL-dependent fatty acid uptake

**Figure 4:**
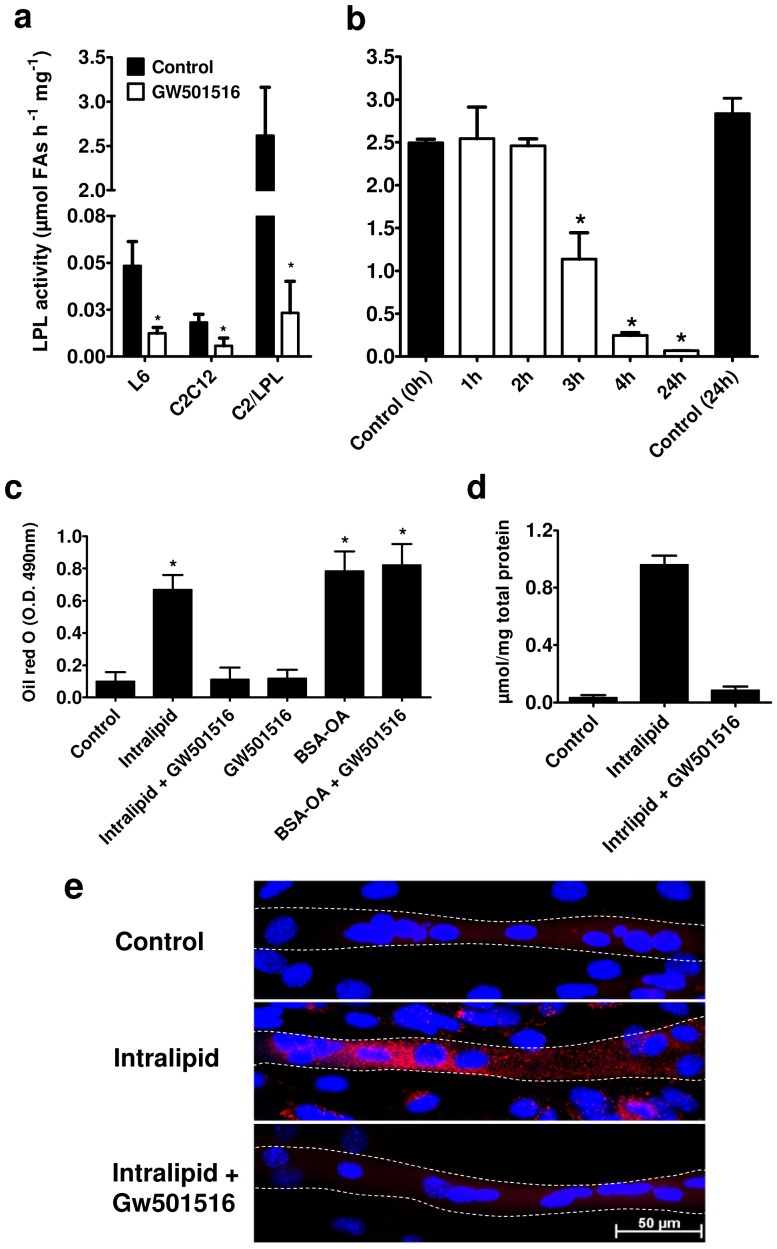
PPARδ activation by GW501516 inhibits LPL activity and LPL-dependent fatty acid uptake. (**a**) Heparin releasable LPL activity was measured in the L6, C2C12 and C2/LPL myotubes after 24 hour incubation of the cells in the presence of GW501516, n = 3. (**b**) Time dependent inhibition of LPL activity by GW501516 (0.1 µM) was measured in heparin releasable pool from C2/LPL myotubes, n = 3. (**c**) Oil Red O staining of myotubes was quantified by densitometry in cells incubated with Intralipid or OA-BSA for 5 hours in the presence or absence of GW501516, n = 4. (**d**) Intracellular triglycerides quantification in cells incubated with Intralipid for 5 hours in the presence or absence of GW501516, n = 3 (**e**) Fluorescence microscope images of C2/LPL myotubes (highlighted with a line) incubated with Intralipid for 5 hours in the presence or absence of GW501516. Nuclei are stained in blue (DAPI) and lipid droplets in red (Oil Red O). * p<0.05, paired *t* test (**a**) or One-way ANOVA with Dunnett's post test (**b, c, d**) compared with Control.

We further evaluated if activation of PPARδ by GW501516 modulates LPL activity in myotubes. L6, C2C12, and C2/LPL myotubes incubated with GW501516 for 24 hours had a significantly lower heparin releasable LPL activity compared with DMSO treated cells (Fig. 4a). Using our methods LPL activity could not be measured in human myotubes. To determine the time dependency of GW501516-mediated LPL inhibition, we incubated C2/LPL cells up to 24 hours and measured the heparin releasable LPL activity. As shown in Fig. 4b LPL activity was decreased by 50% already after 3 hours incubation with GW501516. GW501516 treatment of C2/LPL cells robustly increased Angptl4 mRNA levels already at 1 hour of treatment but had no effect on human LPL mRNA levels, or directly on LPL activity as measured in human post-heparin plasma incubated for one hour with 50 µM GW501516 (Fig. S2 a and b, respectively). Preincubation of C2/LPL myotubes with GW501516 for 20 hours blocked the cellular FA uptake during incubation with Intralipid as measured by Oil Red O staining of the myotubes and as verified by triglyceride quantification in cell lysates (Fig. 4c, d, e) but had no effect on the uptake of OA-BSA (Fig. 4d).

### RXR activation increases Angptl4 expression and inhibits LPL activity in C2/LPL myotubes

**Figure 5:**
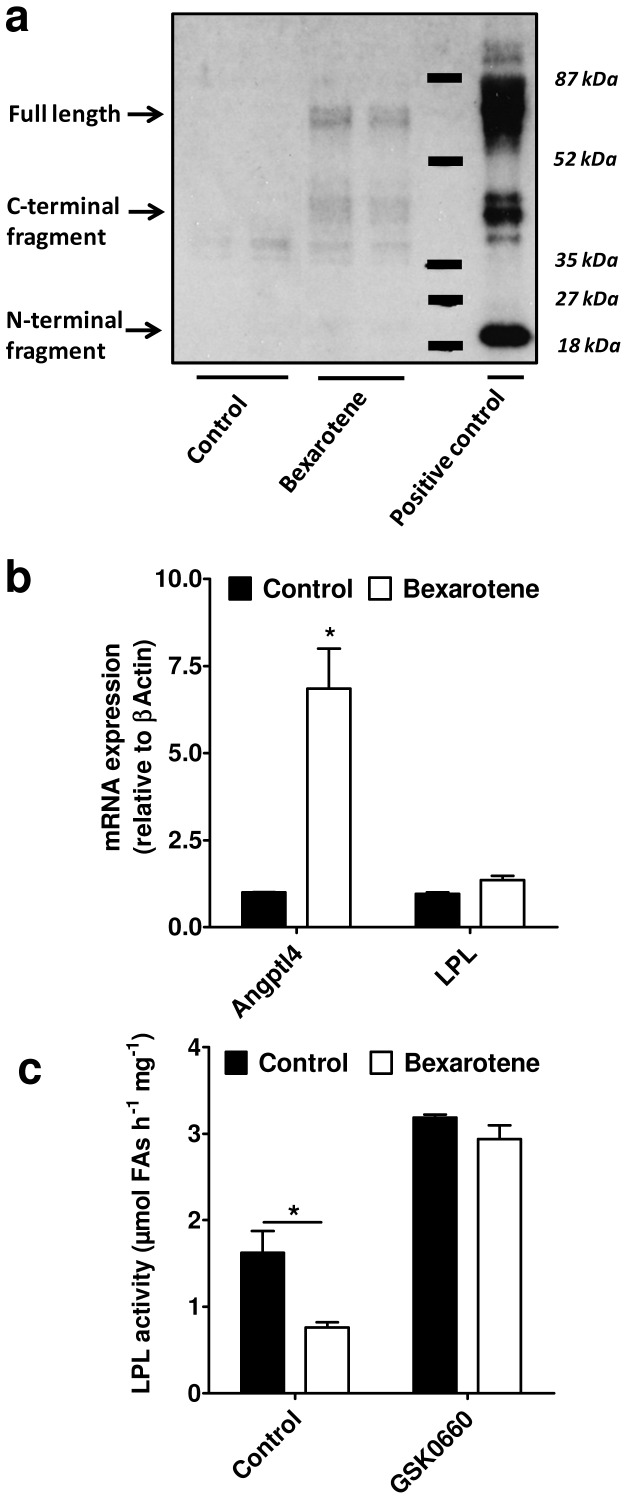
Regulation of Angptl4 expression and LPL activity by bexarotene in C2/LPL myotubes. (**a**) Secretion of Angptl4 from C2/LPL myotubes was analyzed by Western blot 48 hours after incubation with DMSO (Control) or 0.2 µM bexarotene. Samples from two experiments were loaded on the gel. (**b**) Angptl4 and LPL mRNA levels were measured by real time PCR in C2/LPL myotubes incubated with DMSO (Control) or 0.2 µM bexarotene for 24 hours. Values are expressed relative to mouse 36B4 mRNA levels. (**c**) C2/LPL myotubes untreated or pre-treated overnight with 1 µM GSK0660, a PPARδ antagonist, were incubated with DMSO (Control) or 0.2 µM bexarotene for 4 hours. Heparin releasable LPL activity was measured and normalized to the protein content. * p<0.05, Two-way ANOVA with Bonferroni post-tests.

Because PPARδ exerts its function by forming an obligatory heterodimer with retinoic X receptor (RXR) we evaluated whether RXR activation by bexarotene has similar effect compared with that of GW501516 on Angptl4 expression and LPL activity. Indeed, treatment of C2/LPL myotubes with bexarotene strongly induced Angptl4 mRNA and protein levels without significant changes in LPL expression levels as evaluated by real time PCR (Fig. 5a, b). Similarly to GW501516, bexarotene significantly inhibited heparin releasable LPL activity after 4 hours treatment of C2/LPL cells (Fig. 5c). Inhibition of LPL activity by pharmacological activation of RXR was dependent on PPARδ function since incubation of C2/LPL cells with a PPARδ antagonist, GSK0660, completely blocked the bexarotene effect (Fig. 5c).

### Angptl4 mediates PPARδ/RXR effect on LPL activity and LPL-dependent fatty acid uptake

**Figure 6:**
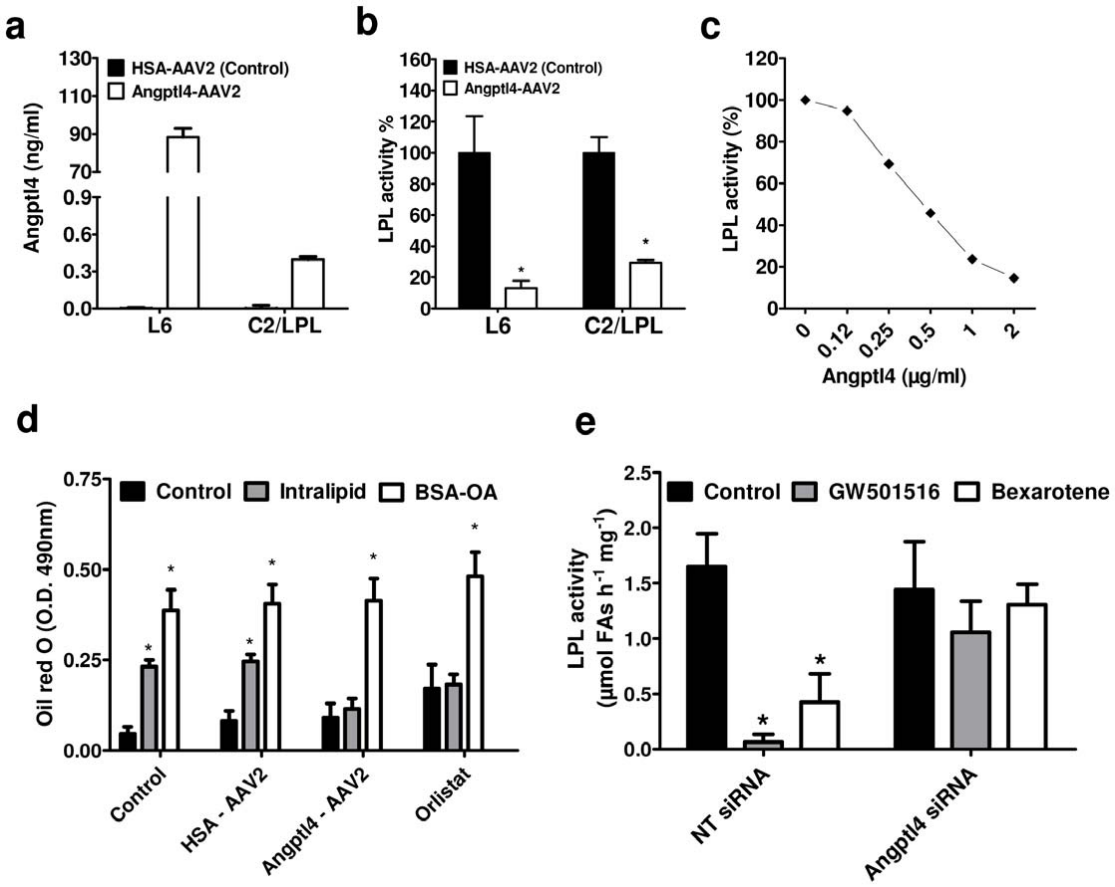
Angptl4 inhibits LPL activity and LPL-dependent fatty acid uptake and mediates PPARδ effect on LPL activity. (**a**) Human Angptl4 concentration was evaluated by ELISA in medium from myotubes infected with HSA (human serum albumin)-AAV2 (Control) or hAngptl4-AAV2. (**b**) Heparin releasable LPL activity was measured in myotubes infected with HSA-AAV2 (Control) or hAngptl4-AAV2, n = 4 for L6 and n = 3 for C2/LPL. LPL activity expressed as 100% represent 0.037 (L6) or 3.38 (C2/LPL) µmol FAs h^−1^ mg^−1^. (**c**) Heparin releasable LPL activity was measured in C2/LPL myotubes exposed to increasing concentration of recombinant hAngptl4. LPL activity expressed as 100% represent 2.58 µmol FAs h^−1^ mg^−1^. (**d**) Myotubes infected with HSA-AAV2 or Angptl4-AAV2 or treated with Orlistat (50 µM) were incubated 16 h with Intralipid or OA-BSA. Oil Red O staining of myotubes was quantified by densitometry, n = 4. (**e**) Cells transfected with non targeting siRNA (NT-siRNA) or Angptl4 siRNA were incubated with GW501516 for 4 hours and heparin releasable LPL activity was quantified, n = 3. * p<0.05, paired *t* test (**b**) or Two-way ANOVA with Bonferroni post-tests (**d, e**) compared with Control.

The direct role of Angptl4 in mediating LPL inhibition by GW501516 and bexarotene was verified using AAV2 and AAV9 encoding hAngptl4, as well as using recombinant hAngptl4 and siRNA targeted silencing. AAV9 delivery of hAngptl4 in mice was performed as described in Methods S1 and resulted in the appearance of hAngptl4 in serum (Fig. S3a) and a concomitant increase in plasma triglycerides (Fig. S3b) when compared with the AAV9 delivery of HSA. Following the Angptl4-AAV2 transduction, the levels of secreted hAngptl4 were moderate in L6 myotubes and very low in C2/LPL myotubes (Fig. 6a). Overexpression of hAngptl4 inhibited LPL activity in both L6 and C2/LPL myotubes when compared with HSA-AAV2 transduced cells (Fig. 6b). When the C2/LPL myotubes were incubated four hours with full length recombinant hAngptl4 we observed a concentration dependent inhibition of LPL activity by hAngptl4 (Fig. 6c). Human Angptl4 or Orlistat (a lipase inhibitor) blocked the LPL-dependent uptake of FAs from Intralipid but had no effect on the uptake of OA-BSA (Fig. 6d). Furthermore, silencing of Angplt4 gene with specific siRNA abolished the inhibitory effect of GW501516 and bexarotene on LPL activity in C2/LPL myotubes (Fig. 6e, see also Fig. S4 for silencing efficiency).

### Angptl4 inhibits intracellular LPL activity and co-localizes with LPL

**Figure 7:**
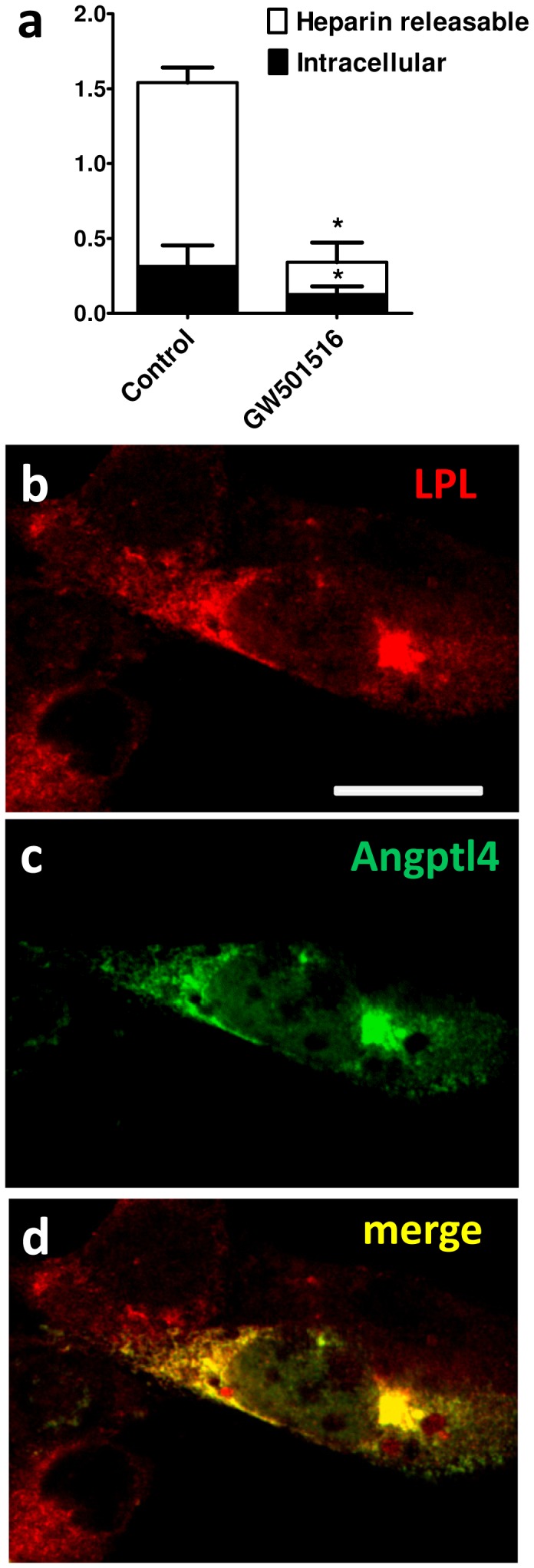
Angptl4 inhibits intracellular LPL activity and co-localizes with intracellular LPL. (**a**) Heparin releasable and intracellular LPL activity was measured in C2/LPL myotubes incubated with GW501516 (0.1 µM) for 24 hours, n = 3. (**b, c, d**) Confocal microscopy of myoblasts transfected with V5 tagged Angptl4 and stained with antibodies against LPL (**b**) and V5 tag (**c**). Scale bar: 20 µm. * p<0.05, paired *t* test.

Because Angptl4 and LPL are produced in the same cells we evaluated inhibition of LPL activity and co-localization of these two factors intracellularly. Although GW501516 treatment inhibited LPL activity mainly in the heparin releasable pool a significant inhibition of LPL activity was also observed intracellularly (Fig. 7a). Intracellular localization of LPL (Fig. 7b) and Angptl4 (Fig. 7c) was confirmed using confocal microscopy. Overlaying of the immunostaining patterns revealed an extensive intracellular co-localization between Angptl4 and LPL (Fig. 7d). The specificity of the antibodies against V5 epitope and LPL was demonstrated using mock transfected cells or via competition approach by pre-incubation of the antibody with exogenous LPL (Fig. S5).

### Angptl4 has no effect on fatty acid oxidation and glucose metabolism

**Figure 8:**
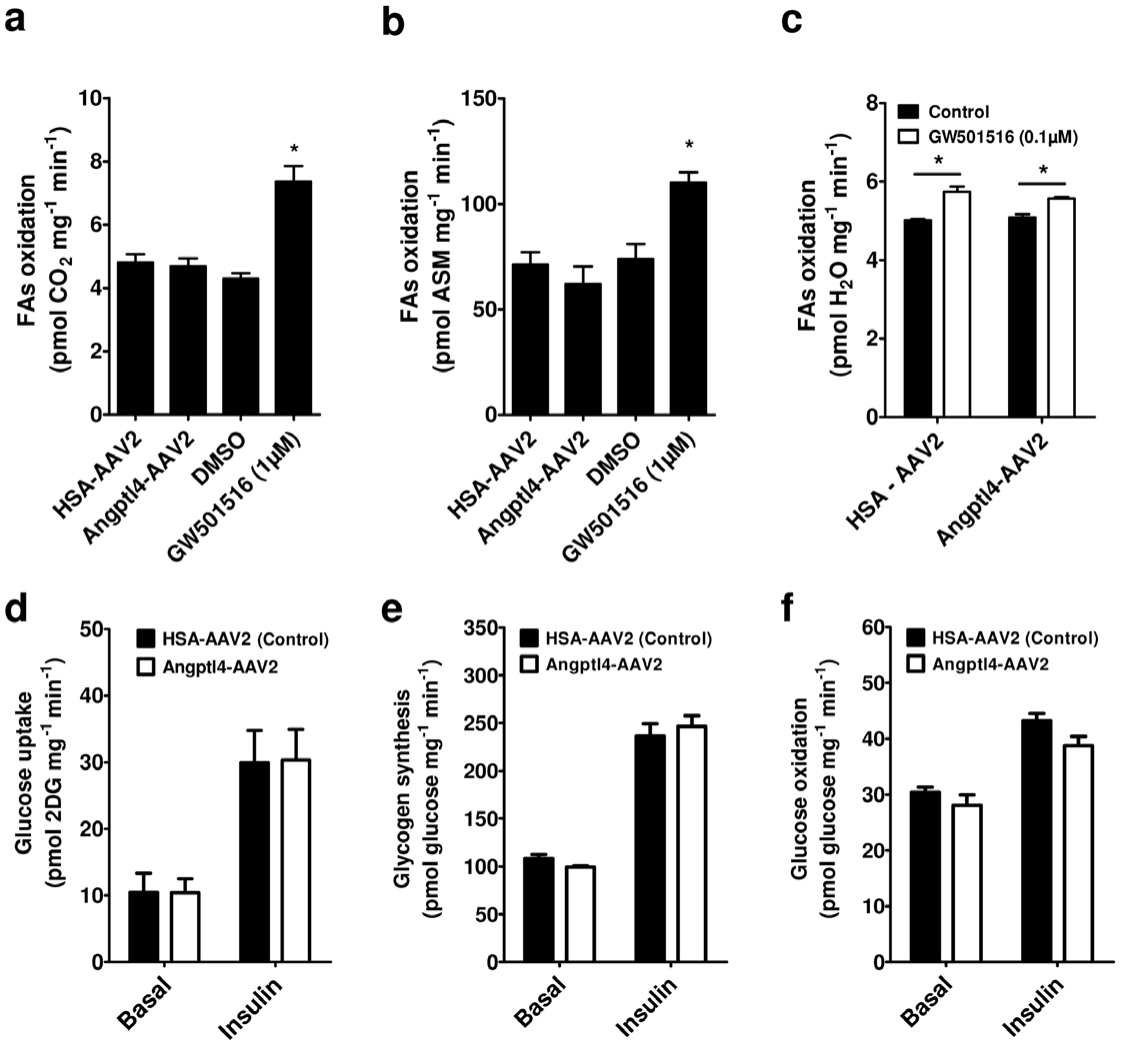
Angptl4 overexpression has no effect on fatty acids oxidation and glucose metabolism in L6 myotubes. Fatty acid (palmitate) oxidation was evaluated by measuring (**a**) ^14^C-CO_2_ or (**b**) ^14^C-acid soluble metabolites (^14^C-ASM) production in L6 myotubes 72 hours post-infection with HSA-AAV2 (Control) or Angptl4-AAV2 and compared with cells incubated with DMSO (Control) or GW501516 for 24 hours, n = 3. (**c**) Palmitate oxidation was evaluated by measuring ^3^H-H_2_O released from myotubes incubated with low doses of GW501516 in the setting of low (HSA-AAV2) and high (Angptl4-AAV2) Angptl4 levels, n = 4. (**d**) Glucose uptake, (**e**) glycogen synthesis and (**f**) glucose oxidation were measured in L6 myotubes 72 hours post-infection with HSA-AAV2 or Angptl4-AAV2 in the absence (Basal) or presence of insulin (100 nM), n = 3. *p<0.05, One-way (**a, b**) or Two-way (**c–f**) ANOVA with Bonferroni post-tests.

Using L6 myotubes we evaluated the role of Angptl4 in the regulation of FA oxidation and glucose metabolism. In contrast to GW501516, Angptl4 overexpression had no effect on palmitate oxidation as verified by ^14^C-CO_2_ and ^14^C-ASM production (Fig. 8a, b). Furthermore, Angptl4 did not change basal and GW501516-induced palmitate oxidation to ^3^H-H_2_O (Fig. 8c). Angptl4 overexpression had no effect on basal and insulin stimulated glucose uptake, oxidation and incorporation into glycogen (Fig. 8d, e, f).

## Discussion

In the present study we have demonstrated that activation of PPARδ/RXR strongly upregulates Angptl4 causing the inhibition of LPL activity and LPL-dependent FA uptake in myotubes. In addition, we have examined the role of Angptl4 in fatty acid oxidation and glucose metabolism.

It was recently reported that in skeletal muscle cells the gene with the highest fold-induction by FAs is Angptl4 and this effect is mediated by PPARδ [11]. Here we confirm a robust increase of Angptl4 by FAs or GW501516, a specific PPARδ activator [26], in both human and mouse myotubes. Processing of endogenous Angptl4 in myotubes showed a similar pattern with that observed in HEK293 or Huh7 cells overexpressing Angptl4 [21], [27], the full length, C-terminal and N-terminal forms being secreted from C2/LPL myotubes treated with GW501516. Angptl4 is a well established LPL inhibitor [12], [13] suggesting that PPARδ activation by FAs can inhibit LPL activity in myotubes. Indeed, treatment of rat and mouse myotubes with GW501516 induced a significant reduction in heparin releasable LPL activity without significant changes in LPL mRNA levels. Kinetic studies in C2/LPL cells showed a robust upregulation of Angptl4 expression already at 1 hour and a 50% reduction of LPL activity at 3 hours after the start of GW501516 treatment. Consequently, the uptake of FAs from Intralipid, which is dependent of LPL activity, was abolished by GW501516. In contrast, the uptake of free fatty acids was not affected by GW501516. Inhibition of LPL activity by PPARδ activation was dependent on Angptl4 mRNA expression since Angptl4 silencing using siRNA blocked the GW501516 effect. Previous studies have shown that in skeletal muscle LPL overexpression increases intracellular triglyceride-pool [28] whereas LPL deletion decreases intracellular triglycerides stores [29]. The LPL-mediated effect on lipid uptake has consequences on insulin signaling cascade in skeletal muscle [28], [29]. Since LPL activity generates FAs, it is possible that activation of the PPARδ-Angptl4 axis functions as a negative feedback mechanism that may serve to protect the muscle fibers from lipid overload (Fig. 7). The mechanism we suggest here is also relevant *in vivo*, i.e. data provided by Tanaka et al. [6] demonstrate that administration of GW501516 to mice fed a high-fat diet markedly reduced the lipid droplet formation in skeletal muscle [6]. The same mechanism was recently described to protect the heart and macrophages from lipotoxicity in mice fed a high-fat diet [30], [31].

In order to regulate gene expression PPARδ forms an obligatory heterodimer with RXR [32]. Bexarotene is a potent and selective RXR agonist that is currently used in the treatment of cutaneous T cell lymphoma and has promising effects in other forms of cancer or dermatologic disorders but its use in clinics is limited due to its undesirable increase effect on plasma triglycerides [33]. The mechanism underlying this side effect is not completely understood. Interestingly, a study performed in rats suggests that bexarotene-induced hypertriglyceridemia is attributable to inhibition of LPL activity in the muscle [34]. This is further supported by the increased skeletal muscle lipoprotein lipase activity observed in RXRγ deficient mice [35]. Here we found that in myotubes the inhibition of LPL activity by bexarotene is dependent on PPARδ and Angptl4. Upregulation of Angptl4 expression by bexarotene is in agreement with previous observations in thyroid cancer cell lines [36]. These data suggest a mechanism that could contribute to increased triglycerides in patients during bexarotene therapy.

Population studies suggest that Angptl4 is not involved in systemic inhibition of LPL in humans [21], [37]. Since Angptl4 is the only known LPL inhibitor expressed in the same cells where LPL is produced, it is conceivable that it has more subtle roles at the tissue level. Overexpression of Angptl4 in myotubes resembles the GW501516 effect on LPL activity and LPL-dependent FA uptake. Angptl4 acts in a concentration dependent manner and interestingly, it appears more effective when expressed together with LPL within the same cells than when added as a recombinant protein. A weaker effect of Angptl4 on LPL activity was also seen in cells treated with conditioned medium from myotubes overexpressing Angptl4 (data not shown). This phenomenon can be explained by our observation that Angptl4 inhibited LPL activity not only at the cell surface, represented by the heparin releasable pool, but also intracellularly. Moreover, confocal microscopy analysis revealed extensive intracellular co-localization of LPL and Angptl4 in reticular perinuclear-concentrated ER membranes. All these observations suggest that LPL and Angptl4 could interact intracellularly making the inhibition more efficient when both proteins are expressed within the same cell. The inhibition of LPL by Angptl4 occurs before GPIHBP1 interaction with LPL and its transport to the luminal site of the capillary endothelium [3], [4]. This notion is physiologically relevant since GPIHBP1 was shown to prevent LPL inhibition by Angptl4 [38]. Interestingly, insulin increases Angptl4 expression in myotubes which is in contrast with adipocytes where insulin downregulates Angptl4 mRNA expression [39]. All these data suggest that Angptl4 could be implicated in the tissue specific regulation of LPL activity by insulin [40], [41].

PPARδ overexpression or activation by GW501516 increased the catabolism of FAs by upregulation of genes implicated in FA uptake, handling, and mitochondrial import in skeletal muscle [5]–[10]. Because Angptl4 is a multifunctional protein we tested whether it plays a role in fatty acid (palmitate) oxidation. As expected, PPARδ activation increased ^14^C-CO_2_ and ^14^C-ASM production by L6 myotubes, however, Angptl4 overexpression had no effect on the β-oxidation. We confirmed this by using ^3^H-palmitic acid and quantification of the ^3^H-H_2_O produced by L6 myotubes overexpressing Angptl4. Angptl4 was suggested to upregulate lipolytic enzymes such as hormone sensitive lipase in C2C12 myocytes [11] and therefore we tested wheather Angptl4 could enhance the oxidative capacity of GW501516. Using a low dose of GW501516 we observed a similar induction of palmitate oxidation in control and Angptl4 overexpressing L6 myotubes. These data strongly suggest that the induction of FA oxidation by PPARδ is not dependent on extra- or intracellular Angptl4.

Previous studies have shown that Angptl4 significantly decreased hepatic glucose production and enhanced insulin-mediated inhibition of gluconeogenesis suggesting a role of Angptl4 in glucose metabolism [42]. In our study, Angptl4 overexpression in L6 myotubes neither affected glucose uptake, glycogen synthesis and glucose oxidation, nor insulin function in regulating these processes. These results suggest a tissue specific regulation of glucose metabolism by Angptl4.

A limitation to consider in our study is that we used cultured myotubes and this system lacks the endothelial cell layer where LPL exerts its function in vivo. Because Angptl4 and LPL are produced in the same cells it is relevant that their interaction should occur before LPL reaches endothelial cells. For this purpose myotubes provide a relevant model to study the mechanism of LPL inhibition by Angptl4 in contrast to liver derived inhibitors that probably act mainly at the luminal surface of the capillary endothelium.

**Figure 9:**
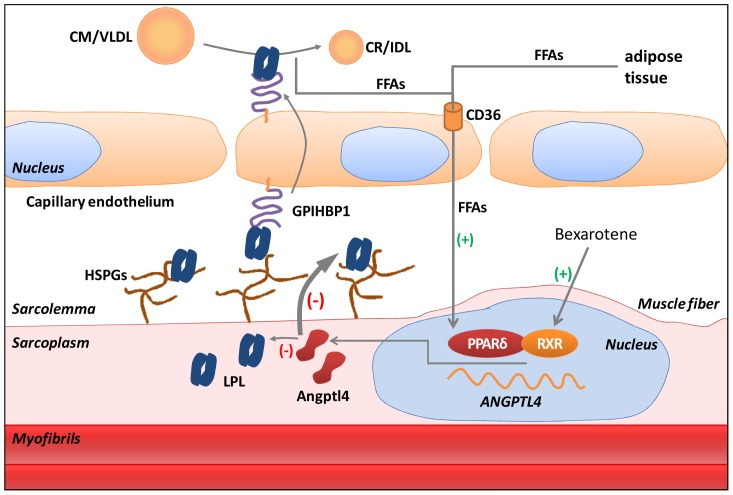
Proposed mechanism regulating LPL activity in the skeletal muscle. The working hypothesis is that FAs produced locally by LPL-mediated hydrolysis of VLDL and chylomicrons (CM) together with FAs derived from adipose tissue (FA-albumin) can activate PPARδ/RXR heterodimer which in turn upregulates Angptl4 gene expression. Angptl4 inhibits LPL activity mainly at the surface of the sarcolemma where less LPL will be available to be transported at luminal sites via the function of GPIHBP1. LPL inhibition by Angptl4 occurs to a lesser extent also intracellularly. This mechanism may protect the skeletal muscle from lipid overload and insulin resistance but may also contribute to bexarotene induced systemic hypertriglyceridemia.

In conclusion, our principal results suggest that an overflow of FAs inhibits LPL activity in skeletal muscle. The working hypothesis is that FAs produced locally via the LPL function or released from adipose tissue as albumin bound FAs can activate PPARδ/RXR heterodimer which in turn upregulates Angptl4 gene expression. Angptl4 inhibits LPL activity mainly at the surface of the sarcolemma where less LPL will be available to be transported at luminal sites via the function of GPIHBP1 and therefore the flow of FAs in the tissue is reduced (Fig. 9). This mechanism may also contribute to increased plasma triglycerides frequently observed in patients treated with bexarotene. In addition, PPARδ increases the FA oxidation capacity of the tissue independently of Angptl4. All these suggest PPARδ as being a key player in maintaining the balance between the FA uptake and oxidation capacity of the skeletal muscle.

## Supporting Information

Figure S1 **Angptl4 mRNA during differentiation of human myotubes.** Angptl4 and PBGD mRNA levels were measured by real time PCR on day 2, 5 and 7 of differentiation in myoblasts derived from four men. Values are expressed as fold increase relative to day two of differentiation and normalized to human βActin mRNA levels, mean±SEM, n = 4. * p<0.05, Student's t test.
(PDF)

Figure S2 **Effect of GW501516 on Angptl4 and LPL mRNA in C2/LPL cells and LPL activity in human post heparin plasma.** (**a**) Angptl4 and LPL mRNA levels were measured by real time PCR in C2/LPL myotubes incubated with DMSO (Control) or GW501516 (0.1 µM) for 1–4 hours. Values were normalized to m36B4 levels and expressed as mean ± SEM, n = 3. (**b**) LPL activity was measured in human post-heparin plasma was incubated with DMSO or GW501516 (50 µM) for 1 hour at RT. * p<0.05, Student's t test.
(PDF)

Figure S3 **Mice were injected with HSA-AAV9 (n = 3) or Angptl4-AAV9 (n = 3) into tibialis anterior muscles and intraperitoneally.** Two weeks after injections plasma (**a**) Angptl4 levels and (**b**) triglycerides were measured by ELISA and enzymatic colorimetric assay respectively. Values are expressed as mean ± SEM of 3 animals per group. * p<0.05, Student's t test.
(PDF)

Figure S4 **Efficiency of mAngptl4 gene silencing in C2/LPL cells.** Mouse Angptl4 mRNA levels were measured by real time PCR in C2/LPL myotubes transfected with non targeting siRNA (NT-siRNA) or Angptl4 siRNA. Values are normalized to HPRT and expressed as mean ± SEM, n = 3.
(PDF)

Figure S5 **Specificity of Angptl4 and LPL immunostainings.** C2/LPL myoblasts were either transfected with the empty vector (mock, **a**) or with Angptl4-V5 (Angptl4, **b**) and immunostained using anti-V5 mAb, FITC Conjugate. Cells were stained with anti-LPL immunopurified IgY that was either preincubated with bovine LPL (+ bovine LPL, **c**) or without bovine LPL (− bovine LPL, **d**). DAPI was used to stain the nuclei (blue). Wide-field fluorescence images were acquired with using Axioplan 2 Imaging E (Zeiss) microscope. Scale bar: 50 µm.
(PDF)

Table S1 **Primers utilized for Real Time PCR.**
(DOCX)

Methods S1 **Supplementary methods for experiments performed in mice.**
(DOCX)
