# Supplementary figures and images for: Angiopoietin-Like 4 Mediates PPAR Delta Effect on Lipoprotein Lipase-Dependent Fatty Acid Uptake but Not on Beta-Oxidation in Myotubes
Source: PLoS One. 2012 Oct 4;7(10):e46212. doi: 10.1371/journal.pone.0046212 (PMC3464237; doi:10.1371/journal.pone.0046212)

Fig. S1

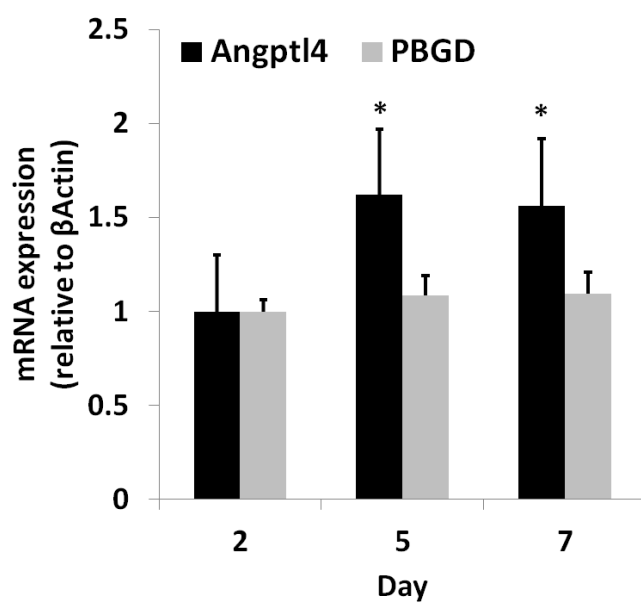

Supplement: Figure S1 — Angptl4 mRNA during differentiation of human myotubes. Angptl4 and PBGD mRNA levels were measured by real time PCR on day 2, 5 and 7 of differentiation in myoblasts derived from four men. Values are expressed as fold increase relative to day two of differentiation and normalized to human βActin mRNA levels, mean±SEM, n = 4. * p<0.05, Student's t test. (PDF) [file pone.0046212.s001.pdf]

Fig. S2

**a**

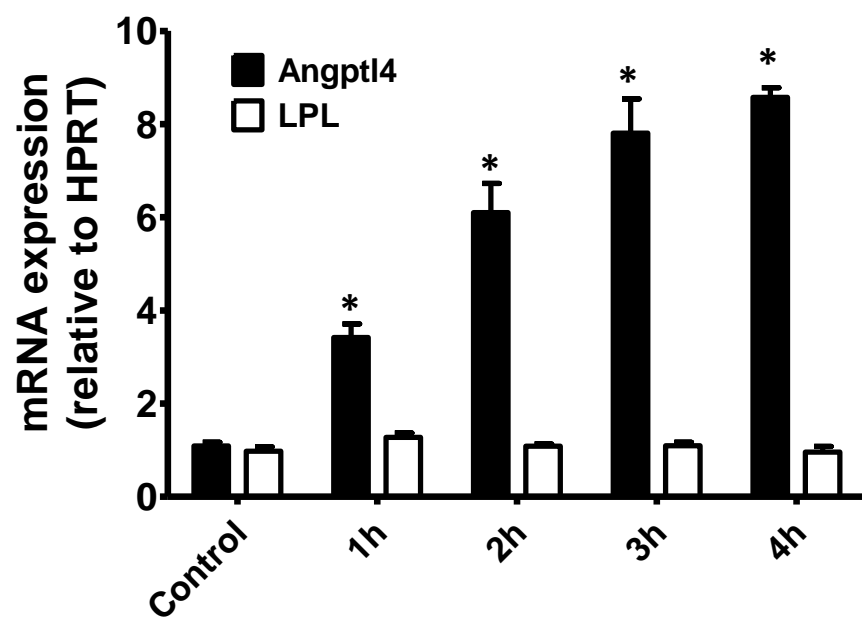

**b**

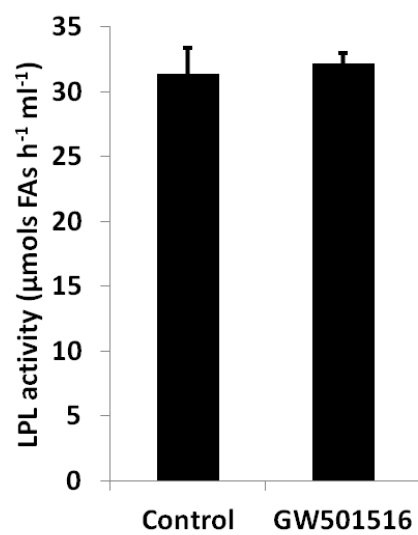

Supplement: Figure S2 — Effect of GW501516 on Angptl4 and LPL mRNA in C2/LPL cells and LPL activity in human post heparin plasma. (a) Angptl4 and LPL mRNA levels were measured by real time PCR in C2/LPL myotubes incubated with DMSO (Control) or GW501516 (0.1 µM) for 1–4 hours. Values were normalized to m36B4 levels and expressed as mean ± SEM, n = 3. (b) LPL activity was measured in human post-heparin plasma was incubated with DMSO or GW501516 (50 µM) for 1 hour at RT. * p<0.05, Student's t test. (PDF) [file pone.0046212.s002.pdf]

Fig. S3

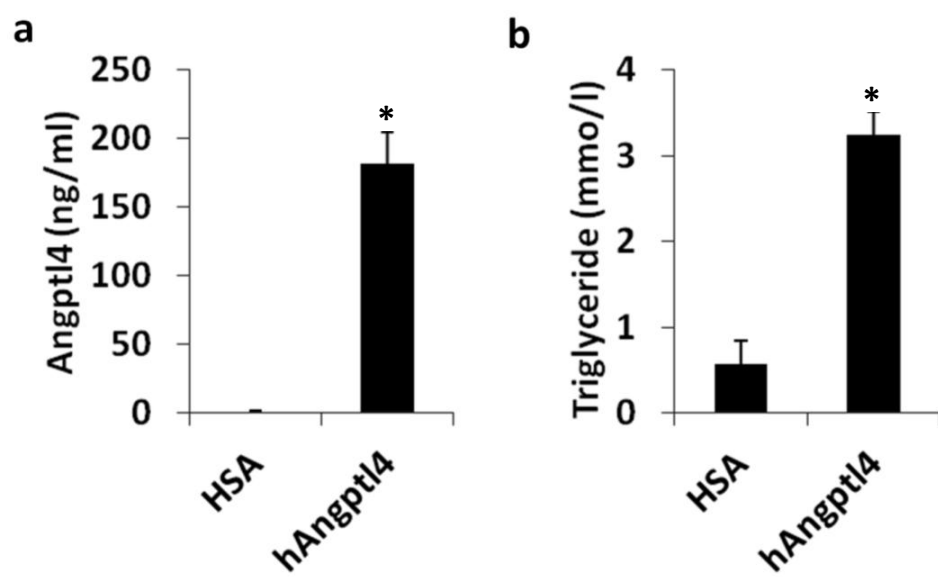

Supplement: Figure S3 — Mice were injected with HSA-AAV9 (n = 3) or Angptl4-AAV9 (n = 3) into tibialis anterior muscles and intraperitoneally. Two weeks after injections plasma (a) Angptl4 levels and (b) triglycerides were measured by ELISA and enzymatic colorimetric assay respectively. Values are expressed as mean ± SEM of 3 animals per group. * p<0.05, Student's t test. (PDF) [file pone.0046212.s003.pdf]

Fig. S4

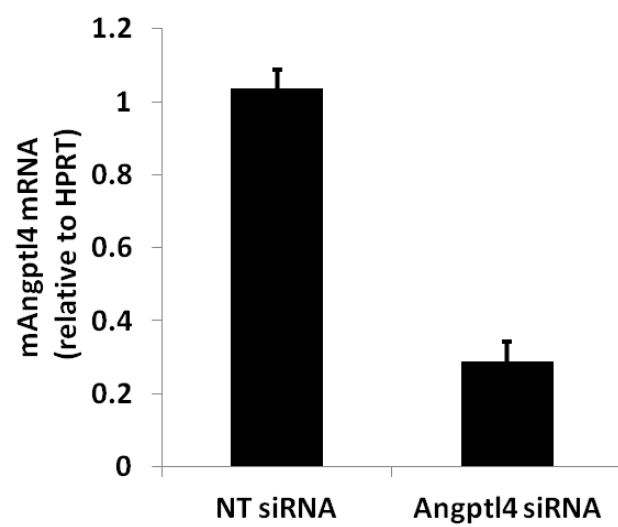

Supplement: Figure S4 — Efficiency of mAngptl4 gene silencing in C2/LPL cells. Mouse Angptl4 mRNA levels were measured by real time PCR in C2/LPL myotubes transfected with non targeting siRNA (NT-siRNA) or Angptl4 siRNA. Values are normalized to HPRT and expressed as mean ± SEM, n = 3. (PDF) [file pone.0046212.s004.pdf]

Fig.S5

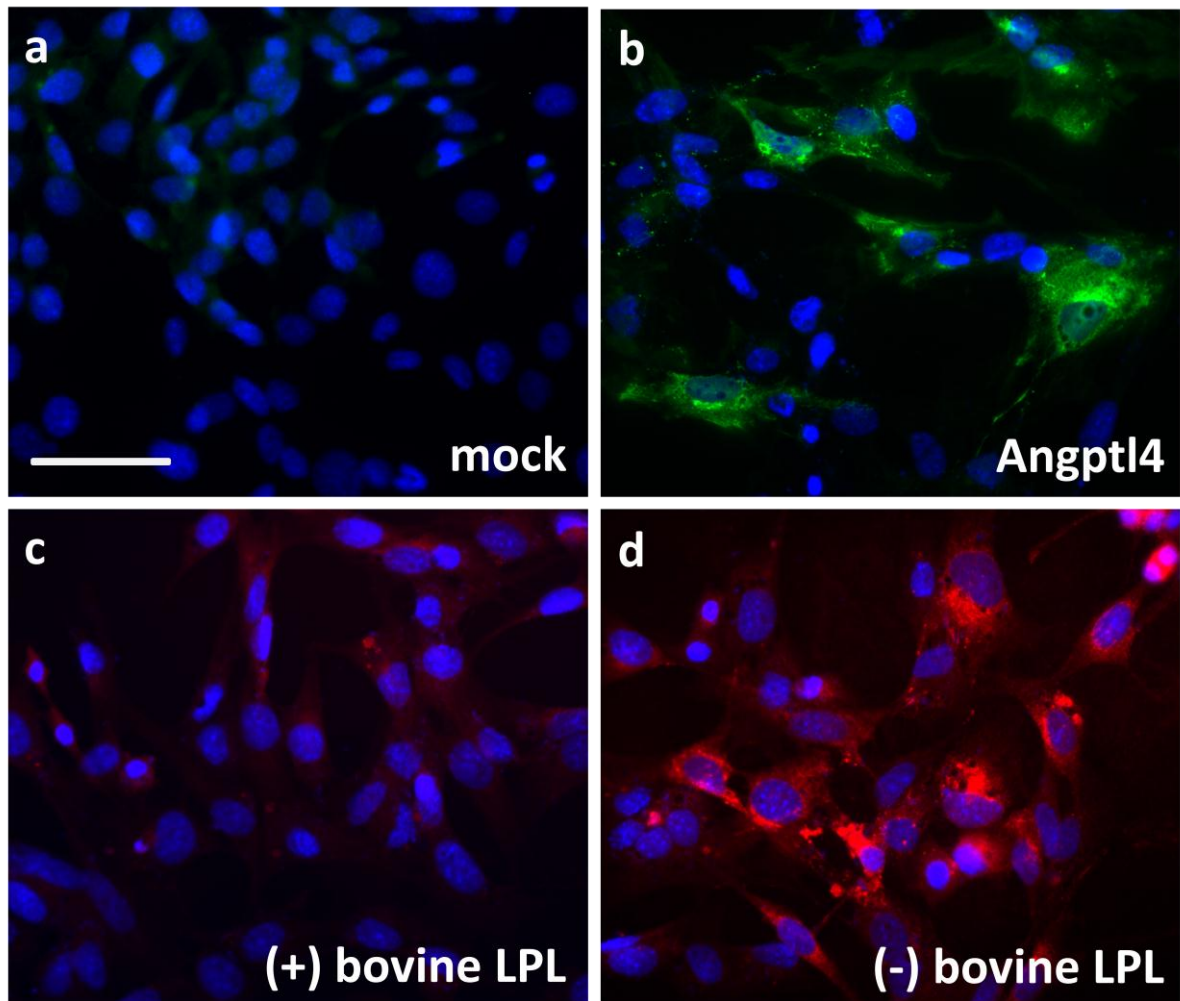

Supplement: Figure S5 — Specificity of Angptl4 and LPL immunostainings. C2/LPL myoblasts were either transfected with the empty vector (mock, a) or with Angptl4-V5 (Angptl4, b) and immunostained using anti-V5 mAb, FITC Conjugate. Cells were stained with anti-LPL immunopurified IgY that was either preincubated with bovine LPL (+ bovine LPL, c) or without bovine LPL (− bovine LPL, d). DAPI was used to stain the nuclei (blue). Wide-field fluorescence images were acquired with using Axioplan 2 Imaging E (Zeiss) microscope. Scale bar: 50 µm. (PDF) [file pone.0046212.s005.pdf]
