# Supplementary material for: Angiopoietin-Like 4 Mediates PPAR Delta Effect on Lipoprotein Lipase-Dependent Fatty Acid Uptake but Not on Beta-Oxidation in Myotubes
Source: PLoS One. 2012 Oct 4;7(10):e46212. doi: 10.1371/journal.pone.0046212 (PMC3464237; doi:10.1371/journal.pone.0046212)
Supplement: Table S1 — Primers utilized for Real Time PCR. (DOCX) [file pone.0046212.s006.docx]

**Table S1** – PCR primers

| Species | Gene | Forward | Reverse |
| --- | --- | --- | --- |
| human | Angptl4 | 5'- GCCTATAGCCTGCAGCTCAC -3’ | 5'- CAAGTGGAGAAGGGTACGGA -3’ |
| human | LPL | 5'- ATTACTGGCCTCGATCCAGCTGG -3’ | 5'- TCCACCAGTCTGACCAGCGGAA -3’ |
| human | Beta Actin | 5'- GATGTGGATCAGCAAGCAGGA -3’ | 5'- AGCATTTGCGGTGGACGAT -3’ |
| human | PBGD | 5'- CATCAGCCTGGCCAACTTGTT -3’ | 5'- TCGTTAAGCTGCCGTGCAA -3’ |
| mouse | Angptl4 (ref 11) | 5'- CAAAACAGCAAGATCCAGCA -3' | 5'- TTGGAAGAGTTCCTGGCAGT -3' |
| mouse | 36B4 | 5'- GGACCCGAGAAGACCTCCTT -3’ | 5'- GCACATCACTCAGAATTTCAATGG -3’ |
| mouse | HPRT | 5'- TTGCTCGAGATGTCATGAAGGA -3’ | 5'- AGCAGGTCAGCAAAGAACTTATAG -3’ |
| mouse | PPARδ (ref 30) | 5’- TTGAGCCCAAGTTCGAGTTTG -3’ | 5’- CGGTCTCCACACAGAATGATG -3’ |
