# Supplementary material for: Angiopoietin-Like 4 Mediates PPAR Delta Effect on Lipoprotein Lipase-Dependent Fatty Acid Uptake but Not on Beta-Oxidation in Myotubes
Source: PLoS One. 2012 Oct 4;7(10):e46212. doi: 10.1371/journal.pone.0046212 (PMC3464237; doi:10.1371/journal.pone.0046212)
Supplement: Methods S1 — Supplementary methods for experiments performed in mice. (DOCX) [file pone.0046212.s007.docx]

**Suplementary methods**

*Mice*

All mouse experiments were approved by the Provincial State Office of Southern Finland and carried out in accordance with institutional guidelines. Institutional animal ethics committee was not necessary because all animal experiments performed in Finland are authorized by the national Animal Experiment Board (Eläinkoelautakunta, ELLA). Six to seven-week-old male BALB/c mice (n=6) were purchased from Charles River Laboratories International, Inc. and housed in individually ventilated cages (IVC). Animals were fed normal chow diet and provided standard temperature, humidity and light/dark conditions for three weeks before experiments. The health status of the animals was checked daily for any suffering symptoms and no signs of pain have been observed during the study.

Mice were anesthetized with xylazine (Rompun, Bayer)-ketamine (Ketalar, Pfizer), and injected with 18 x 10^9^ particles of HSA-AAV9 (n = 3) or Angptl4-AAV9 (n = 3) into tibialis anterior muscles and 6 x 10^10^ AAV9 particles intraperitoneally. Two weeks after injections bloods samples were collected via cardiac puncture following a lethal dose of anesthetics given to the mice. Human Angptl4 and triglycerides levels were quantified in plasma by ELISA and enzymatic colorimetric assay (Cobas, Roche/Hitachi) respectively.
